# Prevalence of Locomotive Syndrome and Its Association with Physical Activity, Frailty, and Cognitive Status Among Community-Dwelling Older Adults in Thailand

**DOI:** 10.3390/ijerph23040414

**Published:** 2026-03-25

**Authors:** Chadapa Rungruangbaiyok, Charupa Lektip, Jiraphat Nawarat, Eiji Miyake, Keiichiro Aoki, Hiroyuki Ohtsuka, Yasuko Inaba, Yoshinori Kagaya, Weeranan Yaemrattanakul

**Affiliations:** 1 Department of Physical Therapy, School of Allied Health Sciences, Movement Science and Exercise Research Center, Walailak University, Nakhon Si Thammarat 80160, Thailand; chadapa.bn@wu.ac.th (C.R.); charupa.le@wu.ac.th (C.L.); nsuparoe@wu.ac.th (J.N.); 2 Department of Rehabilitation, School of Nursing and Rehabilitation Sciences, Showa Medical University, Yokohama-shi 226-8555, Kanagawa, Japan; e.miyake@nr.showa-u.ac.jp (E.M.); k.a-0525@cmed.showa-u.ac.jp (K.A.); ohtsuka@nr.showa-u.ac.jp (H.O.); inaba@nr.showa-u.ac.jp (Y.I.); kagaya@nr.showa-u.ac.jp (Y.K.); 3 Department of Physical Therapy, Faculty of Medicine, Prince of Songkla University, Songkhla 90110, Thailand

**Keywords:** locomotive syndrome, physical activity, transportation-related activity, frailty, cognitive frailty

## Abstract

**Highlights:**

**Public health relevance—How does this work relate to a public health issue?**

- It shows a high prevalence of locomotive syndrome (74.1%) among community-dwelling older adults in Thailand.
- It links daily mobility patterns to physical functioning, connecting mobility with population ageing.

**Public health significance—Why is this work of significance to public health?**

- The study identifies transportation-related physical activity (habitual walking/cycling) as a protective, actionable domain.
- It demonstrates that LS overlaps with but is distinct from frailty and cognitive decline, refining targets for screening.

**Public health implications—What are the key implications or messages for practitioners, policy makers and/or researchers in public health?**

- Routine LS screening must be prioritized, and active-transport strategies should be promoted to preserve mobility in older adults.
- Walkable environments and community mobility programs should be developed.

**Abstract:**

This cross-sectional study included 112 community-dwelling older adults aged ≥ 60 years residing in Tha Sala District, Nakhon Si Thammarat Province, Thailand, recruited using a community-based quota sampling approach. Locomotive syndrome (LS) was assessed using the two-step test and classified according to the Japanese Orthopaedic Association criteria. Physical activity was evaluated using the Thai version of the Global Physical Activity Questionnaire across work-related, transportation-related, and recreational domains. Frailty and cognitive status were assessed using the Thai version of the FRAIL questionnaire and the Montreal Cognitive Assessment, respectively. Binary logistic regression analysis was used to examine associations. The prevalence of LS was 74.1%, with 37.5%, 33.0%, and 3.6% in participants classified as having LS stages 1, 2, and 3, respectively. Transportation-related physical activity was significantly associated with lower odds of LS. Frailty and mild cognitive impairment frequently coexisted with LS but were not independently associated with LS after adjustment for age and sex. Transportation-related physical activity emerged as a key protective factor, highlighting the importance of habitual mobility in daily life. Our findings suggest that LS overlaps with, but is not identical to, frailty and cognitive decline in relatively robust community settings. Early screening and mobility-related physical activity may be crucial in preventing functional decline in rapidly aging societies.

## 1. Introduction

Population aging is accelerating worldwide, particularly in Asia, leading to a growing burden of age-related functional decline and disability [1,2,3,4]. Mobility impairment is a major contributor to loss of independence and is associated with an increased risk of falls, fractures, and long-term healthcare utilization among older adults. Therefore, musculoskeletal disorders have become a critical public health concern in super-aged societies, because of their impact on individual quality of life and their substantial socioeconomic costs, including fracture-related morbidity and healthcare expenditures [5,6,7,8].

In this context, locomotive syndrome (LS) was proposed by the Japanese Orthopaedic Association (JOA) as a clinical concept describing reduced mobility resulting from impairment of the locomotive organs, including bones, joints, muscles, and intervertebral discs [5,9]. Epidemiological evidence from Japan has consistently indicated that LS is highly prevalent among middle-aged and older adults [10]. Using the revised JOA criteria (2020), the Nagahama Study reported that among community-dwelling adults aged ≥ 60 years, the prevalence of LS stages 1, 2, and 3 was 24.4%, 5.5%, and 6.5%, respectively, with a marked increase in prevalence with advancing age [11]. Similarly, a large cross-sectional study involving 35,059 Japanese adults found that 15.0% of participants were classified as LS-positive, with a higher prevalence observed in women than in men [12]. These findings highlight that LS is a common and clinically significant condition in aging populations.

Previous studies have identified several health-related factors and functional characteristics associated with locomotive syndrome (LS). Individuals with LS often demonstrate reduced physical performance, including decreased muscle strength, impaired balance, and slower gait speed, which may contribute to limitations in mobility and activities of daily living [10,11]. In addition, LS frequently coexists with other age-related conditions such as sarcopenia and frailty, suggesting that mobility impairment is closely linked to broader aspects of health status in older adults [3]. Several studies have also reported that musculoskeletal pain, obesity, and metabolic conditions are associated with LS [12]. Furthermore, lifestyle behaviors such as physical inactivity, smoking, and other lifestyle-related factors have been reported to increase the risk of LS in population-based studies [5].

However, most previous studies have primarily focused on individual physical or lifestyle factors, and many were conducted in Japanese populations. Evidence examining the combined relationships between physical activity, frailty, and cognitive status in community-dwelling older adults remains limited, particularly in Southeast Asian settings.

Thailand is currently undergoing a rapid demographic transition into an aged society. According to national statistics, individuals aged ≥ 60 years accounted for 17.8% of the total population in 2020, increasing to 20.0% in 2023 and 20.8% in 2024, indicating that Thailand has already entered an aged society and is rapidly transitioning toward a super-aged society as population ageing continues to accelerate [13]. This demographic shift is expected to place increasing demands on healthcare systems, particularly in the prevention and management of age-related mobility disorders. However, evidence regarding LS in Thailand remains limited. A cross-sectional study conducted in Nan Province reported that 71.5% of middle-aged and older adults exhibited declining locomotive function based on the two-step test, suggesting a substantially higher prevalence of locomotive impairment than that reported in Japanese cohorts [14]. Factors associated with locomotive organ impairment in that study included lower educational attainment, obesity, comorbidities, and limited walking duration. However, comprehensive data on LS prevalence using standardized diagnostic criteria and its association with broader geriatric syndromes in community-dwelling Thai older adults remain scarce.

Physical activity, frailty, and cognitive impairment are closely interrelated geriatric conditions that may influence the development and progression of LS. Insufficient physical activity is associated with a higher risk of LS [15]. In addition to low activity levels, older adults may also face barriers to engaging in regular exercise, including poor health, fear of injury, lack of social support, or limited knowledge and guidance regarding exercise participation, which may further reduce opportunities for maintaining mobility [16]. Frailty, particularly in the pre-frail stage, represents an early phase of vulnerability preceding overt disability [17,18]. Additionally, cognitive impairment, including mild cognitive impairment (MCI), is linked to reduced physical performance, impaired mobility, and an increased risk of functional decline [19]. Nevertheless, few studies have simultaneously examined the relationships between physical activity, frailty status, cognitive function, and LS, particularly in Southeast Asian populations.

Therefore, we aimed to determine the prevalence of LS among community-dwelling older adults in Thailand and to examine its associations with physical activity, frailty, and cognitive status. Understanding these relationships may provide evidence to support early screening and targeted interventions to prevent mobility decline in rapidly aging societies.

## 2. Materials and Methods

### 2.1. Study Design, Setting, and Period of Data Collection

This cross-sectional study was conducted in community-dwelling older adults in Tha Sala District, Nakhon Si Thammarat Province, southern Thailand. Participants were recruited from three community settings: Tha Sala Municipality, Tha Sung Bon Community, and the Walailak Demonstration Community. These communities represent semi-urban residential areas with established primary healthcare networks and a high proportion of older residents. Data collection was carried out between January and June 2025.

### 2.2. Participants and Sampling Method

The study sample consisted of adults aged ≥ 60 years, including both women and men, who resided in Tha Sala District and provided written informed consent. The study employed a community-based quota sampling approach, with simple random selection used within each community quota. The total sample size was allocated approximately equally across three communities in Tha Sala District to ensure balanced representation. Within each community, a list of eligible community-dwelling older adults aged ≥ 60 years was obtained through local coordinators who maintained registries of older residents. Participants were then selected using simple random sampling by a lottery method, whereby individual names were randomly drawn from the eligibility list until the predefined quota for each community was reached. Selected individuals were invited to participate in the study, and those who agreed and provided written informed consent were enrolled.

**Figure 1:**
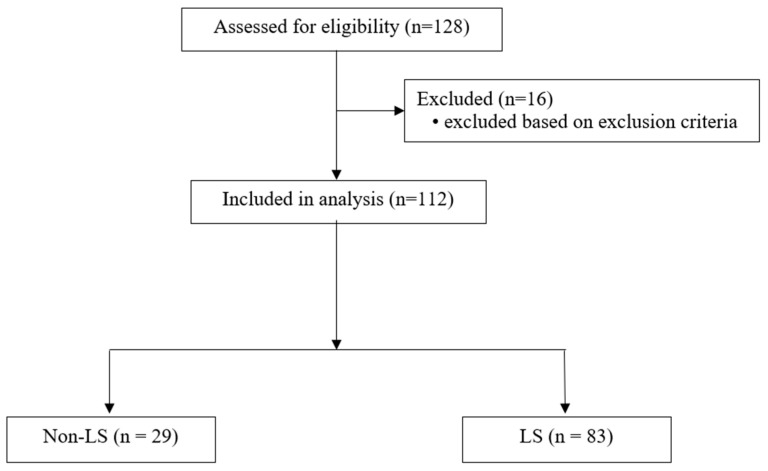
Flowchart of participant recruitment and classification in this cross-sectional study. LS, locomotive syndrome.

Eligible participants were determined as follows: ability to ambulate independently without assistive devices, stand on one leg for at least 10 s on both sides as a pragmatic criterion to ensure safe performance of the physical tests, and communicate in Thai, as well as the presence of complete physical performance tests and questionnaires. Participants were excluded if they had undergone spinal or lower-limb surgery within the previous 3 months, were lower-limb amputees, experienced movement-limiting pain (visual analog scale > 5/10), or developed such pain during testing. Additional exclusion criteria included acute cardiovascular or cerebrovascular disease, severe respiratory disease, clinically diagnosed neurological disorders (e.g., Parkinson’s disease or stroke), vertigo, or dementia, defined as a score < 18 on the Thai version of the Mini-Mental State Examination (MMSE-Thai 2002). Of the 128 individuals screened, 16 were excluded based on the eligibility criteria, resulting in 112 participants included in the final analysis (Figure 1).

### 2.3. Sample Size Calculation

The required sample size was calculated based on the estimated prevalence of LS (71.5% among middle-aged and older adults) reported in a previous Thai study conducted in Nan Province [14]. Sample size estimation was performed using the formula for estimating a population proportion in a cross-sectional study:

$$n=\frac{Z^{2}\times p\times \left(1-p\right)}{d^{2}}$$

where Z = 1.96 for a 95% confidence level, *p* = 0.72, and d = 0.08 (absolute precision). The minimum required sample size was 121 participants. Although the calculated minimum sample size was 121, a total of 112 participants were included owing to feasibility constraints during community recruitment.

### 2.4. Assessment of LS

LS was assessed using the two-step test, a standardized screening tool recommended by the JOA. Participants stood with both feet behind a starting line and were instructed to take two maximal strides forward without losing balance. The total distance covered was measured in centimeters. The test was performed twice, and the longer distance was used for analysis. The two-step test score was calculated by dividing the maximum stride length by the participant’s height [9]. To assess measurement reliability, a subsample of participants (n = 10) was measured twice by the same evaluator, and the intraclass correlation coefficient (ICC) was calculated using a two-way random-effects model. The two-step test demonstrated excellent reliability (ICC = 0.93).

Participants with a two-step test score < 1.3 were classified as having LS. LS severity was further categorized according to the updated JOA criteria as follows: LS stage 1 (<1.3), 2 (<1.1), and 3 (<0.9). For regression analyses, LS was dichotomized as non-LS and LS (stages 1–3) [20]

### 2.5. Assessment of Frailty

Physical frailty was evaluated using the Thai version of the FRAIL questionnaire (T-FRAIL), a validated screening instrument for frailty in older adults [21]. The questionnaire consists of five components: fatigue, resistance, ambulation, illnesses, and weight loss. Each item is scored as 0 or 1, yielding a total score ranging from 0 to 5. Participants were classified as robust (score = 0), pre-frail (score = 1–2), or frail (score ≥ 3) [18]. The questionnaire was administered through interviewer-assisted interviews conducted by trained research staff to ensure that all participants clearly understood the questions. For regression analyses, pre-frail and frail participants were combined into a single group.

### 2.6. Assessment of Cognitive Status and Cognitive Frailty

Cognitive function was evaluated using the Thai version of the Montreal Cognitive Assessment (MoCA-T). MCI was defined as an MoCA score < 25 [22]. For participants with six years of education or fewer, one point was added to the total MoCA score according to the standard scoring procedure of the MoCA-Thai version. Global cognitive screening was performed using the MMSE-Thai 2002 [23]. Participants with MMSE scores < 18 were considered to have dementia and were excluded from the study [24]. Cognitive frailty (CF) was defined as the coexistence of physical frailty (frailty status) and MCI in the absence of diagnosed dementia [24,25].

### 2.7. Physical Activity Assessment

Physical activity was assessed using the Thai version of the Global Physical Activity Questionnaire (GPAQ) [26], which was translated and validated by the Thailand Physical Activity Knowledge Development Center [27]. The questionnaire evaluated physical activity across three domains: work-related, transportation-related, and recreational activities.

Total and domain-specific physical activity were calculated and expressed as metabolic equivalent task (MET)-min per week. For regression analyses, physical activity variables were analyzed as continuous variables per 100 MET-min/week.

### 2.8. Covariates

Demographic and health-related variables included age, sex, body mass index (BMI), educational level, and multimorbidity. Multimorbidity was assessed as the total number of self-reported chronic conditions. For descriptive analyses, multimorbidity was categorized as the presence of ≥2 chronic diseases, consistent with commonly used definitions. For regression analyses, the number of chronic conditions was treated as a continuous variable to evaluate incremental associations with LS.

### 2.9. Statistical Analysis

Descriptive statistics were used to summarize participant characteristics. Normality of continuous variables was assessed using the Shapiro–Wilk test. As most continuous variables were not normally distributed, they were presented as medians with interquartile ranges (IQRs), whereas categorical variables were summarized as frequencies and percentages. Group comparisons between participants with and without LS were performed using the Mann–Whitney U test for continuous variables and the chi-square test or Fisher’s exact test for categorical variables, as appropriate. Associations between LS and demographic factors, physical activity, frailty status, and cognitive status were examined using binary logistic regression analyses. Crude odds ratios (ORs) and 95% confidence intervals (CIs) were obtained from univariable models. Adjusted ORs were estimated using multivariable models controlling for age and sex. In the regression analyses, the reference categories were defined as the lowest-risk groups: robust status for frailty and normal cognitive status for cognitive function. Physical activity variables were analyzed as continuous variables (per 100 MET-min/week), and therefore no categorical cut-off values were applied. Multicollinearity among predictor variables was assessed using variance inflation factors (VIF). All VIF values were below 2, indicating no evidence of problematic multicollinearity. Statistical significance was set at *p* < 0.05. All analyses were conducted using SPSS version 25.0 (IBM Corp., Armonk, NY, USA).

### 2.10. Ethical Considerations

The study protocol was approved by the Human Research Ethics Committee of Walailak University (approval number: WUEC-24-423-01). Written informed consent was obtained from all participants prior to data collection.

## 3. Results

### 3.1. Study Population and Participant Characteristics

A total of 112 community-dwelling older adults were included in the final analysis. The participant recruitment and classification processes are illustrated in Figure 1. Of the total sample, 29 participants were classified as non-LS and 83 as LS. Baseline demographic, health-related, and physical activity characteristics according to LS status are summarized in Table 1. Participants with and without LS did not differ significantly with respect to age, sex, or BMI (all *p* > 0.05). Similarly, the prevalence of multimorbidity, defined as the presence of ≥2 chronic conditions, was comparable between the two groups.

When physical activity was examined by domain, participants with LS demonstrated significantly lower levels of transportation-related physical activity than those without LS (median [IQR]: 2.8 [4.4] vs. 5.6 [5.6] MET-min/100/week, *p* = 0.023). In contrast, no significant differences were observed in total physical activity, work-related physical activity, recreational physical activity, or physical activity level categories between the two groups (all *p* > 0.05). Participants with LS also reported a higher median number of chronic conditions; however, this difference did not reach statistical significance.

### 3.2. Prevalence of LS, Frailty, and Cognitive Impairment

Among the 112 community-dwelling older adults included in the study, 83 participants had LS, yielding an overall prevalence of 74.1%. Regarding LS severity, 42 participants (37.5%) had LS stage 1, 37 (33.0%) LS stage 2, and four (3.6%) LS stage 3. MCI was identified in 99 participants (88.4%) at baseline (Table 1). With respect to frailty status, 59 participants (52.7%) were classified as robust, whereas 44 (39.3%) and nine (8.0%) were classified as pre-frail and frail, respectively.

### 3.3. Factors Associated with LS

The adjusted logistic regression models demonstrated acceptable goodness-of-fit according to the Hosmer–Lemeshow test (all *p* > 0.05). The Nagelkerke pseudo R^2^ values ranged from 0.07 to 0.11, indicating modest explanatory power.

Table 2 presents the crude and adjusted associations between demographic factors, physical activity, and LS. In univariable logistic regression analyses, age, female sex, BMI, and multimorbidity were not significantly associated with LS. Similarly, total, work-related, and recreational physical activity showed no significant associations with LS.

In contrast, transportation-related physical activity was significantly associated with LS. Higher transportation-related physical activity (per 100 MET-min/week) was associated with lower odds of LS in both crude analysis (OR = 0.83, 95% CI: 0.71–0.97, *p* = 0.021) and after adjustment for age and sex (adjusted OR = 0.82, 95% CI: 0.70–0.96, *p* = 0.015). No other physical activity domain demonstrated significant associations with LS after adjustment.

### 3.4. Associations with LS (Cognitive and Frailty Factors)

Associations between cognitive status, frailty status, and LS are presented in Table 3. In crude analyses, participants with MCI tended to have lower odds of LS than those in participants without MCI; however, this association did not reach statistical significance (OR = 0.35, 95% CI: 0.11–1.16, *p* = 0.085). After adjustment for age and sex, the association remained borderline but non-significant (adjusted OR = 0.31, 95% CI: 0.09–1.06, *p* = 0.061).

In contrast, pre-frail or frail participants exhibited higher odds of LS compared with robust participants in crude analysis (OR = 2.51, 95% CI: 1.02–6.15, *p* = 0.045). This association was attenuated after adjustment for age and sex and became marginally non-significant (adjusted OR = 2.47, 95% CI: 1.00–6.14, *p* = 0.052).

## 4. Discussion

### 4.1. Main Findings

This cross-sectional study examined the prevalence of LS and its associations with physical activity, frailty, cognitive status, and selected demographic and health-related factors among community-dwelling older adults in Thailand. The principal findings are as follows: First, the prevalence of LS was high, affecting approximately three-quarters of the study population, indicating a substantial burden of locomotive dysfunction among older adults living independently. Second, among the physical activity domains, transportation-related physical activity was inversely associated with LS, whereas total physical activity and other physical activity domains were not. Third, although frailty and cognitive impairment frequently coexisted with LS, neither condition was independently associated with LS after adjustment for age and sex. Finally, traditional risk factors, including age, sex, BMI, and comorbidities, were not significantly associated with LS in multivariable analyses.

Collectively, these findings highlight the importance of habitual mobility-related activities and suggest that LS represents a functional condition that overlaps with, but remains distinct from, frailty and cognitive impairment.

### 4.2. Prevalence of LS

The present study demonstrated a high prevalence of LS among community-dwelling older adults in Thailand, underscoring LS as a substantial and potentially under-recognized public health concern in rapidly aging societies outside Japan. The observed prevalence of LS in this cohort exceeded that reported in several large-scale Japanese population-based studies, even when early-stage LS was considered.

In Japan, epidemiological data indicate considerable variability in the prevalence of LS, depending on diagnostic criteria and population characteristics. The Nagahama Study, which applied the revised 2020 LS criteria among adults aged ≥ 60 years, reported that 24.4% of participants had LS stage 1, whereas 5.5% and 6.5% had LS stages 2 and 3, respectively, yielding an overall LS prevalence of approximately 36% [11]. These findings suggest that, although early-stage LS is relatively common, advanced LS stages remain less prevalent among community-dwelling older Japanese adults. In contrast, the ROAD study, a population-based cohort involving adults aged ≥ 60 years, demonstrated that when early-stage impairment was included, LS stages 1 and 2 affected more than 80% of participants, whereas frailty and sarcopenia were present in only 4.5% and 8.7% of the population, respectively [28]. Collectively, these findings suggest that LS often precedes overt frailty and disability and may represent an early manifestation of age-related mobility decline.

Evidence from Thailand remains limited; however, available data indicate a substantial LS burden. A cross-sectional study conducted in Nan Province reported that 71.5% of community-dwelling adults aged 50–87 years exhibited declining locomotive function, as assessed using the two-step test [14]. This prevalence is markedly higher than that reported in Japanese cohorts using comparable functional assessments, suggesting that LS may be more prevalent or detected at a later stage in Thai populations. Differences in lifestyle patterns, occupational demands, health literacy, and access to preventive musculoskeletal care may partially explain these discrepancies. However, this comparison should be interpreted with caution because the present study was based on a relatively small community-based sample compared with the large-scale population studies cited above.

The high prevalence of LS observed in this study should also be interpreted within the context of Thailand’s demographic transition. Thailand has recently entered a “complete aged society,” defined as having at least 20% of the population aged ≥ 60 years. National statistics indicate that the proportion of older adults increased from 17.8% in 2020 to more than 20% by 2023, reflecting the rapid pace of population aging [13]. Unlike Japan, where LS screening and early intervention strategies have been systematically promoted by the JOA, Thailand currently lacks structured national programs specifically targeting early mobility decline. Consequently, functional deterioration may accumulate before clinical recognition, contributing to the higher prevalence of LS observed in community settings.

Collectively, these findings suggest that LS is highly prevalent among older adults in Thailand and may manifest earlier or progress more rapidly than in populations with established screening and prevention systems. The contrast in LS prevalence between Thailand and Japan underscores the importance of contextual factors, including demographic change, lifestyle, and healthcare infrastructure, in shaping the burden of mobility-related disorders. Early identification of LS in community-dwelling older adults may therefore represent a critical opportunity to prevent downstream frailty, disability, and loss of independence in aging societies.

### 4.3. Physical Activity and LS

In this study, lower levels of transportation-related physical activity were significantly associated with LS. Each additional 100 MET-min/week of transportation-related physical activity was associated with 17% lower odds of LS in the crude model and 18% lower odds after adjustment for age and sex. This domain-specific association suggests that the type and context of physical activity may be more relevant to locomotive function than overall activity volume alone. Transportation-related activity typically consists of moderate-to-vigorous physical activity (MVPA), such as brisk walking or cycling, performed habitually and reflecting the functional mobility required for independent living. These movement patterns closely resemble the functional demands assessed by LS screening tests, including the two-step and stand-up tests.

These findings are consistent with those of previous lifestyle-focused studies in Japan, which demonstrated that LS is strongly associated with daily functional behaviors, pain, anxiety regarding physical fitness, and self-rated health, rather than with structured exercise alone [29]. Therefore, transportation-related activity may serve as a proxy marker of preserved lower-limb strength, balance, and confidence in ambulation, all of which are central to LS pathophysiology.

The lack of significant associations observed for other physical activity domains may reflect contextual factors specific to older adults in Thailand. Work-related physical activity is limited in this age group because of retirement, resulting in low variability, whereas recreational physical activity is often irregular and less structured, potentially leading to measurement bias when assessed using self-report questionnaires. Additionally, aggregating all domains into total physical activity may dilute the effects of functionally meaningful activities. Similar domain- and intensity-specific patterns have been reported in Japanese populations, where MVPA, but not light-intensity activity, was associated with lower odds of LS, with notable differences according to age and sex [30,31].

Taken together, these findings suggest that promoting transportation-related physical activity, through the development of safe and accessible walking environments, may represent a pragmatic and culturally appropriate strategy for preventing LS among community-dwelling older adults in Thailand.

However, the possibility of reverse causation should also be considered when interpreting this finding. Because the present study used a cross-sectional design, it is possible that older adults with better mobility and physical function are more capable of engaging in transportation-related physical activity, rather than transportation-related activity directly reducing the risk of locomotive syndrome. Therefore, the observed association should be interpreted with caution, and longitudinal studies are needed to clarify the temporal relationship between daily mobility-related physical activity and the development of locomotive syndrome.

### 4.4. Frailty and LS

Although frailty and LS frequently coexisted in the present study, frailty was not independently associated with LS after adjustment for age and sex. This finding supports the concept that LS and frailty are overlapping but distinct geriatric syndromes rather than interchangeable conditions.

Previous population-based studies have demonstrated substantial overlap between these conditions. Imagama et al. reported that approximately 42% of frail individuals met the criteria for LS, highlighting the frequent coexistence of these conditions among community-dwelling older adults [32]. Importantly, this overlap does not imply equivalence, as a considerable proportion of individuals with LS are not frail, suggesting that LS may occur earlier along the continuum of functional decline. Further evidence for a stage-dependent relationship has been reported in community settings. Nishimoto et al. demonstrated a clear stage-dependent association between LS and pre-frailty among community-dwelling older adults, with the prevalence of pre-frailty increasing progressively across LS stages and a significant trend observed after adjustment for demographic and health-related factors [33]. These findings support the idea that worsening locomotive impairment is closely associated with the development of early frailty in community settings.

In community-dwelling populations with a relatively low prevalence of frailty, LS may therefore manifest earlier in the functional decline trajectory, preceding the transition to overt frailty. The absence of an independent association between frailty and LS in the present study may reflect limited statistical power, differences in operational definitions, and the relatively preserved health status of the study population. Clinically, these findings suggest that screening solely for frailty may fail to identify individuals with early locomotive dysfunction who remain robust or pre-frail, underscoring the potential value of incorporating LS screening into routine geriatric assessments.

### 4.5. Cognitive Status and CF

In the present study, neither MCI, as defined by the MoCA cutoff, nor CF was independently associated with LS after adjustment for age and sex. Although cognitive impairment and LS frequently coexisted at a descriptive level, the absence of an independent association suggests that the relationship between mobility decline and cognition may be more complex and context dependent than a direct one-to-one correspondence.

In the present study, a high prevalence of mild cognitive impairment (MCI) was observed (88.4%). This proportion appears higher than that reported in many community-based studies of older adults. Several factors may partly explain this finding.

First, cognitive status in the present study was assessed using the Montreal Cognitive Assessment (MoCA), which is known to be more sensitive than traditional screening tools such as the Mini-Mental State Examination (MMSE) for detecting early cognitive changes and mild cognitive impairment in community populations [22]. Consequently, the use of MoCA may lead to a higher proportion of individuals being classified as having possible MCI compared with studies that rely primarily on MMSE-based screening.

Second, the educational profile of the study population may also have influenced cognitive screening performance. In this study, approximately three-quarters of participants had only primary education or no formal education. Educational attainment has been shown to influence performance on cognitive screening instruments, even when education-adjusted scoring procedures are applied, particularly in community-based older populations.

Third, the present study reflects cognitive screening outcomes rather than clinically diagnosed MCI. Screening instruments such as the MoCA are designed to detect early or subtle cognitive decline and therefore may identify individuals at risk of cognitive impairment rather than those with clinically confirmed MCI. As a result, the observed prevalence should be interpreted as indicating potential cognitive vulnerability within this community-based sample rather than the true prevalence of clinically diagnosed MCI. Similar methodological considerations have been noted in studies examining cognitive frailty and early cognitive decline in older adults [19].

Evidence from community-dwelling populations suggests that the association between LS and cognitive impairment varies according to population characteristics and cognitive assessment methods. In a study of community-dwelling elderly women in Japan, Nakamura et al. reported that individuals with LS, defined using the Geriatric Locomotive Function Scale-25, had significantly higher odds of cognitive impairment assessed by the MMSE, even after adjustment for age [34]. These findings suggest that reduced locomotive function may be associated with global cognitive impairment in specific populations, particularly when broader cognitive screening tools are used.

By contrast, the present study did not identify an independent association between LS and MCI, as defined by the MoCA after adjustment for age and sex. This discrepancy may be attributable to differences in cognitive assessment instruments, sex distribution, and the overall health status of the study population. The MoCA is more sensitive to subtle executive dysfunction, whereas the MMSE primarily reflects global cognitive decline. Collectively, these findings suggest that LS may be more strongly associated with advanced or global cognitive impairment than with early-stage cognitive decline in relatively robust community-dwelling older adults.

The concept of CF further clarifies this relationship. According to the IANA/IAGG consensus and subsequent updates, CF is defined as the coexistence of physical frailty and cognitive impairment in the absence of dementia and is considered a potentially reversible state. Population-based studies indicate that the prevalence of CF is generally low when strict diagnostic criteria are applied, often ranging from approximately 1% to 2% among community-dwelling older adults [19], which may partly explain the lack of an independent association between CF and LS observed in this study.

Overall, these findings suggest that although cognitive impairment and LS may coexist in community-dwelling older adults, their relationship is not necessarily direct or uniform. In relatively robust populations, LS may primarily reflect musculoskeletal and functional decline, whereas cognitive impairment and frailty may emerge later or through partially independent pathways. Clinically, this underscores the importance of multidimensional assessment, as reliance on mobility or cognitive screening alone may fail to identify early vulnerability in other domains. Longitudinal studies using harmonized definitions of cognition and frailty are warranted to clarify the temporal and causal relationships among locomotive dysfunction, cognitive decline, and CF.

### 4.6. Limitations

This study has several limitations. First, the relatively small sample size may have limited statistical power to detect independent associations between LS and demographic or clinical factors. In addition, the final sample size (n = 112) was slightly lower than the initially calculated minimum sample size, which may further affect statistical precision. Second, participants were recruited from communities within a single district in southern Thailand; therefore, the findings may not be fully generalizable to older adults living in other regions or in different sociocultural or environmental contexts. Third, the restricted age range of participants (≥60 years) may have reduced age-related variability and attenuated age effects. Fourth, the cross-sectional design precludes causal inference between LS and associated factors and raises the possibility of reverse causation in the observed associations.

Physical activity was assessed using a self-reported questionnaire, which may be subject to recall bias despite interviewer-assisted administration. In addition, frailty status was also assessed using a questionnaire-based instrument, which may introduce measurement bias. Finally, the two-step test used to assess locomotive syndrome evaluates maximal stride length and overall locomotor performance rather than steady-state gait speed. Therefore, test performance may be influenced by several physical factors, including balance ability, lower-limb muscle strength, and anthropometric characteristics such as body height or leg length. Although the two-step value is normalized by body height to partially account for body size differences, these factors may still contribute to variability in test performance. Moreover, participation required voluntary consent, which may introduce potential selection bias, as individuals who agreed to participate may differ from those who declined participation. These limitations should be considered when interpreting the findings.

## 5. Conclusions

LS was highly prevalent among community-dwelling older adults in Thailand. Transportation-related physical activity was significantly associated with LS, whereas frailty, cognitive impairment, and traditional demographic factors showed partial overlap without independent associations. These findings highlight the potential relevance of habitual mobility-related activities in daily life and support the role of LS as an early indicator of functional decline in aging societies. However, given the cross-sectional design of the study, the observed associations should be interpreted with caution, and longitudinal studies are needed to clarify the temporal relationships between physical activity and LS.

## Figures and Tables

**Table 1:** Baseline characteristics of participants according to LS status.

Characteristic	Total	Non-LS	LS	*p*-Value
	(n = 112)	(n = 29)	(n = 83)	
Age (years), median (IQR)	68 (8)	68 (7)	68 (11)	0.939 ^a^
Sex, n (%)				0.209 ^b^
Male	15 (13.4)	6 (20.7)	9 (10.8)	
Female	97 (86.6)	23 (79.3)	74 (89.2)	
BMI (kg/m^2^), median (IQR)	24.9 (5.7)	24.5 (5.5)	25.0 (6.4)	0.502 ^a^
Educational level, n (%)				0.262 ^b^
Low education (no formal education/primary)	84 (75.0)	24 (82.8)	60 (72.3)	
Higher education (secondary or above)	28 (25.0)	5 (17.2)	23 (27.7)	
Physical activity (per 100 MET-min/week), median (IQR)				
Total physical activity	29.5 (28.4)	30.8 (27.9)	29.2 (27.6)	0.976 ^a^
Work-related physical activity	16.8 (27.6)	16.8 (28.2)	16.8 (25.2)	0.891 ^a^
Recreational physical activity	7.2 (4.8)	5.6 (6.0)	7.2 (4.8)	0.100 ^a^
Transportation-related physical activity	3.6 (3.6)	5.6 (5.6)	2.8 (4.4)	0.023 ^a,^*
Physical activity level, n (%)				0.503 ^b^
Low	2 (1.8)	1 (3.4)	1 (1.2)	
Moderate	55 (49.1)	12 (41.4)	43 (51.8)	
High	55 (49.1)	16 (55.2)	39 (47.0)	
Multimorbidity (≥2 conditions), n (%)	55 (49.1)	14 (48.3)	41 (49.4)	0.311 ^c^
Number of chronic conditions, median (IQR)	1.0 (1.0)	1.0 (2.0)	2.0 (2.0)	0.150 ^a^
LS severity, n (%)				–
Non-LS	29 (25.9)	29 (100.0)	–	–
LS stage 1	42 (37.5)	–	42 (50.6)	
LS stage 2	37 (33.0)	–	37 (44.6)	
LS stage 3	4 (3.6)	–	4 (4.8)	
Mild cognitive impairment (MCI), n (%)				0.095 ^c^
Non-MCI	13 (11.6)	6 (20.7)	7 (8.4)	
MCI	99 (88.4)	23 (79.3)	76 (91.6)	
Frailty status, n (%)				0.059 ^c^
Robust	59 (52.7)	20 (69.0)	39 (47.0)	
Pre-frail	44 (39.3)	9 (31.0)	35 (42.2)	
Frail	9 (8.0)	0 (0.0)	9 (10.8)	
Cognitive frailty (CF), n (%)				0.109 ^c^
Non-CF	103 (92.0)	29 (100.0)	74 (89.2)	
CF	9 (8.0)	0 (0.0)	9 (10.8)	

Notes: Values are presented as median (IQR) or number (%). ^a^ *p*-value based on Mann–Whitney U test. ^b^ *p*-value based on the chi-square test. ^c^ *p*-value based on Fisher’s exact test. Statistically significant associations are indicated by an asterisk (* *p* < 0.05). Abbreviations: BMI, body mass index; MET, metabolic equivalent task; LS, locomotive syndrome; MCI, mild cognitive impairment; CF, cognitive frailty; IQR, interquartile range.

**Table 2:** Associations between demographic factors, physical activity, and LS.

Variable	Crude OR (95% CI)	*p*-Value	Adjusted OR ^†^ (95% CI)	*p*-Value
Demographic and health-related factors				
Age (years)	1.02 (0.95–1.10)	0.510	1.03 (0.96–1.11)	0.443
Sex (Female)	0.47 (0.15–1.45)	0.187	0.45 (0.14–1.40)	0.167
BMI (kg/m^2^)	1.05 (0.95–1.15)	0.332	1.05 (0.95–1.16)	0.326
Multimorbidity (per additional chronic condition)	1.36 (0.93–2.00)	0.110	1.32 (0.89–1.95)	0.165
Physical activity (per 100 MET-min/week)				
Total physical activity	1.01 (0.99–1.02)	0.482	1.01 (0.99–1.02)	0.461
Work-related physical activity	1.01 (0.99–1.02)	0.458	1.01 (0.99–1.02)	0.435
Recreational physical activity	1.07 (0.96–1.19)	0.221	1.07 (0.96–1.19)	0.208
Transportation-related physical activity	0.83 (0.71–0.97)	0.021 *	0.82 (0.70–0.96)	0.015 *

Notes: Values are presented as odds ratios (ORs) with 95% confidence intervals (CIs). Crude ORs were obtained from univariable binary logistic regression analyses. Adjusted ORs ^†^ were obtained from multivariable logistic regression models adjusted for age and sex. Physical activity variables were expressed per 100 MET-min/week. Statistically significant associations are indicated by an asterisk (* *p* < 0.05). Abbreviations: BMI, body mass index; LS, locomotive syndrome; MET, metabolic equivalent of task.

**Table 3:** Associations of cognitive status and frailty status with LS.

Variable	Crude OR (95% CI)	*p*-Value	Adjusted OR ^†^ (95% CI)	*p*-Value
Mild cognitive impairment (yes vs. no)	0.35 (0.11–1.16)	0.085	0.31 (0.09–1.06)	0.061
Pre-frail/Frail (vs. robust)	2.51 (1.02–6.15)	0.045 *	2.47 (1.00–6.14)	0.052

Notes: Values are presented as odds ratios (ORs) with 95% confidence intervals (CIs). Crude ORs were obtained from univariable binary logistic regression analyses. Adjusted ORs ^†^ were obtained from multivariable logistic regression models adjusted for age and sex. Statistically significant associations are indicated by an asterisk (* *p* < 0.05). Abbreviations: LS, locomotive syndrome.

## Data Availability

The data presented in this cross-sectional study are not publicly available due to ethical restrictions and the need to protect participant confidentiality. Data may be made available by the corresponding author upon reasonable request and with approval from the Walailak University Ethics Committee.

## Abbreviations

The following abbreviations are used in this manuscript:

BMI	Body Mass Index
MET	Metabolic Equivalent of Task
LS	Locomotive Syndrome
Non-LS	Non-Locomotive Syndrome
MCI	Mild Cognitive Impairment
CF	Cognitive Frailty
PA	Physical Activity
IQR	Interquartile Range
OR	Odds Ratio
CI	Confidence Interval
JOA	Japanese Orthopaedic Association
GPAQ	Global Physical Activity Questionnaire
MMSE	Mini-Mental State Examination
MoCA	Montreal Cognitive Assessment
MVPA	Moderate-to-Vigorous Physical Activity
